# Evaluation of filtering blebs using the ‘Wuerzburg bleb classification score’ compared to clinical findings

**DOI:** 10.1186/1471-2415-12-24

**Published:** 2012-07-17

**Authors:** Sandra Furrer, Marcel N Menke, Jens Funk, Marc Töteberg-Harms

**Affiliations:** 1 University of Zurich, Medical Faculty, Zurich, Switzerland; 2 Department of Ophthalmology, Bern University Hospital, Bern, Switzerland; 3 Department of Ophthalmology, UniversityHospital of Zurich, Frauenklinikstrasse 24, 8091, Zurich, Switzerland; 4 Massachusetts Eye & Ear Infirmary, Harvard Medical School, Boston, MA, USA

**Keywords:** Filtering bleb, Glaucoma, Filtration surgery, Bleb grading, Trabeculectomy

## Abstract

**Background:**

To determine the agreement between intraocular pressure and the ‘Wuerzburg bleb classification score’, as well as between single items of the score and intraocular pressure. Interobserver variability was analyzed.

**Methods:**

57 post-trabeculectomy eyes were included. Colour photographs were used to score the filtering bleb in accordance to the Wuerzburg bleb classification score by two different examiners. At the same visit, clinical data such as intraocular pressure, best corrected visual acuity, slit lamp biomicroscopy and medical history were obtained by another examiner.

**Results:**

After trabeculectomy, 42 out of 57 eyes (73.7%) reached the target pressure (≤21mmHg, and intraocular pressure reduction of at least 20%, without antiglaucoma medication, and without any additional intervention). Fair agreement was found between intraocular pressure and Wuerzburg bleb classification score ≥8 points and ≥7 points (kappa 0.24 and 0.27, respectively). Analyzing the subgroups of the morphological criteria, best agreement was found between occurrence of microcysts and target intraocular pressure (к 0.22 – 0.34).

**Conclusions:**

Evaluating filtering blebs after trabeculectomy by using the Wuerzburg bleb classification score is a good technique for predicting intraocular pressure control in eyes attaining a minimum score of seven points. The presence of microcysts on the filtering bleb predicts that the eye is likely to attain target pressure.

## Background

Since first described by Cairns in 1968 [1], trabeculectomy (TE) has been widely used and remains the gold standard in surgical treatment of glaucoma. Long-term success depends on preoperative and intraoperative conditions, but long-term success also highly depends on the persistence of filtration efficiency at the bleb site. Therefore, postoperative observation and care of the developing filtering bleb in clinical practice is an important tool in reaching the target pressure after filtration surgery in a higher percentage of the patients [2,3].

Since 1989, a number of observations and classifications of filtering blebs using morphological criteria have been described [4-7]. These early efforts included various assessments of filtering bleb morphology and emphasize a general consensus in the morphologic features that indicate favourable in contrast to unfavourable filtering bleb development [8]. The most frequent matter of failing filtering surgery is still subconjunctival scarring which needs intensified postoperative care of the filtering bleb even if intraocular pressure (IOP) remains well controlled [9]. In order to allow a well-structured assessment of bleb morphology and to predict potential signs of failure early enough (to initiate wound healing modulation therapy), different bleb grading systems including the classification system described by Picht and Grehn [10,11], the Indiana Bleb Appearance Grading Scale (IBAGS) [8] and the Moorfileds Bleb Grading System (MBGS) [12] were published. All of them have been developed as stand-alone grading tools. The ‘Wuerzburg bleb classification score (WBCS)’, based on the bleb classification as described by Picht and Grehn [10,11] was the first standardized grading system and was introduced in a prospective study to investigate the influence of clear cornea phacoemulsification on filtering bleb morphology, function and IOP [13]. The WBCS and its modification is often used as well in recently announced studies [14-17] as in clinical practice and consistent evaluation and documentation is given by satisfactory interobserver variability [18]. The careful clinical bleb evaluation following the WBCS includes the items vascularisation, corkscrew vessels, encapsulation and microcysts. The item height is usually excluded from the score as it may have favourable and unfavourable aspects as the filtering bleb can be prominent either in over filtering or in encapsulated blebs [2]. In a semi quantitative analysis in 1998, Picht and Grehn found out that filtering blebs with favourable outcomes had a higher quantity of microcysts, a lower quantity of conjunctival and corkscrew vessels, a lower prevalence of encapsulation, and a decrease in height compared to filtering blebs with unfavourable outcome [11]. In agreement, Sacu *et al.*[19] demonstrated 2003 that filtering blebs with conjunctival subepithelial microcysts showed a good prognosis whereas corkscrew vessels are associated with a higher risk of encapsulation. Nevertheless, the control of IOP remains the principal goal of all current glaucoma treatment to avoid progression of optic nerve damage [20-22], and therefore the main aim of this study was to analyse the agreement between the morphological appearance of the filtering bleb using the WBCS and the IOP. Furthermore, we looked for agreements between single items of the WBCS and the IOP and we analysed the interobserver variability of the WBCS. In this first study we wanted to evaluate the usability and overall correlation of the WBCS and IOP in clinical practice.

## Methods

In this prospective study, we analyzed 57 eyes of 51 consecutive patients who had undergone primary fornix-based TE with intraoperative Mitomycin C (MMC, 0.25mg/ml) between February 2007 and September 2009 by one experienced glaucoma specialist (JF). The application time of MMC varied between 2.5 minutes and 3.5 minutes. Diagnoses were as follows: Primary open-angle glaucoma (42%), pseudoexfoliative glaucoma (42%), normal pressure glaucoma (7%), chronic angle-closure glaucoma (3.5%), juvenile glaucoma (3.5%) and dysgenetic changes in the chamber angle (2%). The study was performed at the UniversityHospital Zurich, Switzerland and was approved by the local Ethics committee. It was conducted adhering to the tenets of the Helsinki Declaration and in compliance with all local and national regulations and directives. After trabeculectomy all patients of the defined study period were invited to participate in this prospective study. Signed informed consent was obtained prior to first study related examination.

**Figure 1:**
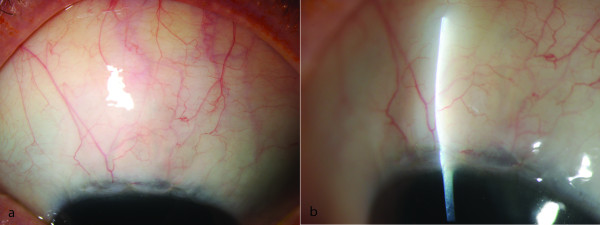
**(a) overview, (b) slitlamp photograph: Filtering bleb with WBCS = 11 points (Vascularisation = 2, Corkscrew vessels = 3, Encapsulation = 3, Microcysts = 3).** IOP postoperatively = 13 mmHg, no anticlaucoma medication, no additional secondary intervention. a) overview b) slit lamp photograph.

**Figure 2:**
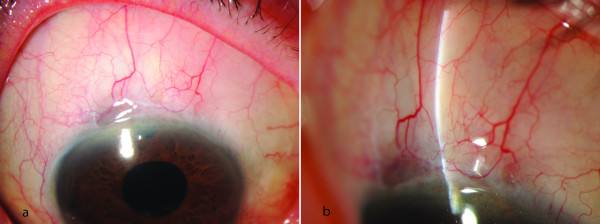
**(a) overview, (b) slitlamp photograph: Filtering bleb with WBCS = 6 points (Vascularisation = 2, Corkscrew vessels = 3, Encapsulation = 1, Microcysts = 0).** IOP postoperatively = 18 mmHg, no antiglaucoma medication, 1 additional secondary intervention (cyclophotocoagulation). a) overview b) slit lamp photograph.

All study patients were examined once after trabeculectomy between April 2009 and March 2010. The study Data recorded were gender, age at the time of TE, eye, glaucoma diagnosis and postoperative secondary interventions. In addition, use of anti-hypertensive medication was noted. Ophthalmic examinations were performed between 16 and 1007 days after operation (mean 536 ± 226 days) by one examiner (SF) and consisted of IOP, best corrected visual acuity and slit lamp biomicroscopy. The IOP was measured with Goldmann applanation tonometry [23]. The tonometer (AT 900, Haag-Streit AG, Könitz, Switzerland) was calibrated by the manufacturer periodically. At the same visit, slit-lamp photographs were obtained of every filtering bleb in order to enable subsequent masked grading of morphological bleb appearance by two examiners (JF and MT-H), with differing levels of clinical experience. Bleb photography included an overview in a standardized fashion as well as slit-lamp perspective through the area of maximal bleb elevation at an angle of 45 degrees (Figures 1 and 2). The detailed grading and scoring of the photographs was conducted following the WBCS criteria, which includes the items vascularisation, corkscrew vessels, encapsulation and microcysts (Table 1). Most parameters are scored from 0 to 3. The height of the bleb was noted separately using multiples of corneal thickness. It is based on the highest point from the scleral surface to the bleb. The WBCS is considered to be used in daily clinical practice by ophthalmologists with different levels of experience. The filtering bleb pictures were evaluated by the two raters. One was well trained the other had less experience. It was not possible to grade the microcysts on the photographs in most cases. The presence or absence of microcysts was evaluated and graded at the same time as the clinical examination by the glaucoma specialist (JF) using the slit-lamp, as it is not possible to grade microcysts in every part of the filtering bleb solely from photographs. They were scored per third of the bleb area containing microcysts.

**Table 1:** WBCS

**Parameters**	**Scoring**
Vascularity	3 = avascular
	2 = similar to adjacent conjunctiva
	1 = increased
	0 = massive
Corkscrew vessels	3 = none
	2 = in one third
	1 = in two thirds
	0 = entire bleb
Encapsulation	3 = none
	2 = in one third
	1 = in two thirds
	0 = entire bleb
Microcysts	3 = entire bleb
	2 = lateral or medial of the flap
	1 = over the scleral flap
	0 = none

When evaluating treatment outcomes, long-term IOP control was classified as ‘successful’ if IOP postoperatively did not exceed 21 mmHg and was reduced by at least 20% of the preoperative level without any antiglaucoma medication and without any additional intervention, e.g. cyclophotocoagulation and re-trabeculectomy (target IOP). The preoperative IOP level was defined as that measured at the last clinical visit prior to surgery. The filtering bleb was classified as ‘successful’ if the bleb attained a WBCS of at least 10 points as suggested by Klink *et al.*[13]. In a second approach, we noted the numbers of ‘successful blebs’ when defining success as attaining a WBCS of 9, 8, 7, 6 or 5 points. The WBCS scores included in the analysis where those obtained by our experienced glaucoma specialist (JF). The results of the bleb grading undertaken by the second practitioner (MT-H) were noted separately.

Statistical analysis was performed using Excel for Windows (Microsoft Office 2003, Microsoft Corp., WA, USA) and PASW statistics software (version 18.0.0, SPSS Inc. Chicago, IL, USA). In order to calculate the agreement between our two definitions of success we used cross-classified tables with к-values. For interpreting levels of agreement, the following guidelines were employed: к = 0: no agreement better than chance, к < 0.20: poor strength of agreement, к = 0.21 - 0.40: fair strength of agreement, к = 0.41 - 0.60: moderate strength of agreement, к = 0.61 - 0.80: good strength of agreement, к = 0.81 - 1.00: very good strength of agreement.

To reflect the clinical reproducibility of the WBCS, the consistency and absolute agreement of the two graders was calculated with the intraclass correlation coefficient (ICC) using a 2-way random model. Levels of agreement that were > 0.81 were defined as excellent agreement, levels between 0.61 and 0.80 were defined as good agreement, levels between 0.41 and 0.60 were defined as moderate agreement, levels between 0.21 and 0.40 were defined as fair agreement and levels less than 0.21 were defined as poor agreement [18,24]. Systematic biases as well as limits of agreement were evaluated by using Bland-Altman plots in MedCalc Statistical software (version 11.3.6, MedCalc Software bvba, Mariakerke, Belgium, data not shown).

## Results

In 57 eyes of 51 consecutive patients (31 left eyes, 26 right eyes), a fornix-based TE with intraoperative MMC was performed. 20 patients were men and 31 were women. The mean age of the patients was 67.6 ± 11.8 years (30.2-84.1 years) on the day of surgery. Sixteen patients (16 eyes) were unable to complete all of the required follow-up examinations. In 11 patients this was due to unwillingness to attend the clinic in order to complete the examinations, three further patients lived overseas, whilst two more patients were unable to attend during the study period because of pre-existing health problems. Mean follow-up period was 536 ± 226 days (16–1007 days) post intervention. Mean number of IOP-lowering drugs decreased from 2.5 ± 1.4 preoperatively to 0.3 ± 0.8 postoperatively on the day of study visit. In 49 eyes (85.96%) IOP lowering treatment could even be stopped, while it could be at least lowered in 5 eyes (8.77%). Only three eyes (5.26%) needed the same number of antiglaucoma medicines as before. After TE the mean IOP decreased by 10.8 mmHg compared to the mean IOP prior surgery; mean IOP preoperatively was 24.0 ± 6.5 mmHg compared to 13.2 ± 7.3 mmHg postoperatively.

On the day of the ophthalmic examination, 44 (77.2%) eyes were found to achieve an IOP of less than or equal to 21 mmHg and an IOP reduction of at least 20% compared to the preoperative level, without antiglaucoma medication. 42 eyes (73.7%) reached the target pressure. Using the gradings of our experienced glaucoma specialist (JF), 25 eyes reached a WBCS score of ≥10 points (43.9%). Thus, comparing these two definitions of success, more eyes were classified as successful based on IOP (73.7%) rather than WBCS (43.9%). Further, 36 eyes attained a score of ≥9 points (63.2%), 47 eyes ≥8 points (82.5%), 48 eyes ≥7 points (84.2%) and 53 eyes both ≥6 and ≥ 5 points (93%).

Table 2 shows the agreement between target pressure and different scores of the WBCS. There is a poor agreement between target IOP and WBCS of ≥10 points (к 0.17), ≥9 points (к 0.20), ≥6 points and ≥5 points, respectively (к 0.11). Nevertheless, fair agreement was found for WBCS scoring of ≥8 points and ≥7 points (к 0.24 and 0.27, respectively). Therefore, the best agreement was found between target IOP and WBCS of at least 7 points. From the 25 eyes reaching a WBCS of ≥10 points, 21 also reached the target pressure (84%).

**Table 2:** Cross table target pressure vs. different scores including value of agreement

		**Score ≥ 10**		**Score ≥ 9**		**Score ≥ 8**		**Score ≥ 7**		**Score ≥ 6**		**Score ≥ 5**	
		**No**	**Yes**	**No**	**Yes**	**No**	**Yes**	**No**	**Yes**	**No**	**Yes**	**No**	**Yes**
Target IOP reached	No	11	4	8	7	5	10	5	10	2	13	2	13
	Yes	21	21	13	29	5	37	4	38	2	40	2	40
Agreement	Kappa		.17		.19		.24		.27		.11		.11

Regarding agreement between single items of the WBCS and target pressure, best agreement was found between occurrence of microcysts and target IOP reached (к 0.22 – 0.34, Table 3) and between extent of vascularity and target IOP not reached (к 0.31, Table 4). The more microcysts that exist on the filtering bleb, the better is the agreement with reaching the target pressure. In contrast, we found poor strength of agreement between the IOP and the factors of cork screw vessels and encapsulation.

**Table 3:** Cross table target pressure vs. microcysts including value of agreement

		**Microcysts 3 points**		**Microcysts ≥ 2 points**		**Microcysts ≥ 1 point**	
		**No**	**Yes**	**No**	**Yes**	**No**	**Yes**
Target IOP reached	No	14	1	8	7	6	9
	Yes	25	17	9	33	4	38
Agreement	Kappa		.22		.31		.34

**Table 4:** Cross table target pressure vs. vascularization including value of agreement

		**Vascularization ≤ 1 point**	
		**No**	**Yes**
Target IOP not reached	No	39	3
	Yes	10	5
Agreement	Kappa		.31

The agreement between the WBCS and IOP using the results of the grader with less clinical experience (MTH) were as follows: poor agreement between target IOP and WBCS of ≥10 points, ≥9 points, ≥7points, ≥6 points and ≥5 points (к 0.12, 0.095, 0.149, 0.169, 0.026) and fair agreement between target IOP and WBCS of ≥8 points (к 0.219).

Table 5 shows the ICC between the two raters. Consistency and absolute agreement of the two raters were: vascularisation +0.77/+0.73, corkscrew vessels +0.73/0.72, encapsulation +0.49/+0.49, bleb height +0.87/+0.85 and bleb score without microcysts +0.63/+0.61. By using Bland-Altman plots, systemic bias could be excluded for the factors corkscrew vessels and encapsulation. Vascularization and bleb score without microcysts were scored higher by the experienced glaucoma specialist while the item bleb height was scored higher by the grader with less clinical experience.

**Table 5:** Bleb morphology graded from photographs: consistency and absolute agreement between the two raters

	**Consistency**	**Absolute agreement**
Vascularization	0.77	0.73
Corkscrew vessels	0.73	0.72
Encapsulation	0.49	0.49
Bleb height	0.87	0.85
Score without microcysts	0.63	0.61

Limits of agreements (evaluated by Bland-Altman plots): vascularization +1.41/-0.81, corkscrew vessels +1.48/-1.17, encapsulation +2.4/-2.0, bleb height +1.21/-1.88, bleb score without microcysts +3.0/-2.6.

## Discussion

The main aim of our prospective study was to investigate whether there is an agreement between postoperative IOP and the WBCS. Klink *et al.* established a semi-quantitative score and classified the filtering blebs before and after clear cornea phacoemulsification as ‘favourable’ when they reached a WBCS of 10 points or more, and ‘unfavourable’ if less than 10 points were scored [13]. Using this classification of ‘success’ of bleb morphology as detectable with slit lamp biomicroscopy, we found only poor agreement to our defined target IOP (Table 2). But after expanding the definition of ‘success’ on the morphological findings, we found fair agreement between the attainment of at least 7 points (к 0.273) and our target IOP (Table 2). Based on these findings we conclude that the WBCS is applicable in clinical practice. It is very interesting to compare these results with those published by Klink *et al*. Even though they did not find any prognostic value of the early WBCS bleb score for the long-term success of TE (20% pressure reduction with reference to the pretreated IOP level and an upper IOP limit of 21 mmHg without glaucoma medication), they found that bleb score of more than 8 points in the first two weeks after TE seemed to be associated with a lower IOP (≤12 mmHg) 1 year postoperatively, while patients with a total bleb score of less than 7.0 two weeks postoperatively showed a higher IOP [25]. Our study compares the WBCS and a defined target IOP at the same time, but nonetheless we found a similar ‘cut off’ on the WBCS scale. The fact that in our study 21 out of 25 eyes reaching a WBCS of ≥ 10 points also achieved the target IOP (84%) indicates that the WBCS works well in predicting IOP control when at least ten points are attained.

In addition, we looked for agreements between single items of the WBCS and the postoperative IOP. We found a fair agreement between the occurrence of microcysts and reaching the target pressure. This is in accordance with Picht *et al.*[11,16] and Sacu *et al.*[19]. In contrast, our data reveal that excessive vascularisation shows a fair agreement to higher IOP postoperatively but we found no agreement between the presence of corkscrew vessels and IOP, nor between presence of bleb encapsulation and IOP.

Our study strengthens the clinical utility of the WBCS with good interobserver consistency, and absolute agreement in assessment of the factors vascularisation, corkscrew vessels and bleb score (excluding microcysts). The item bleb height was evaluated with excellent consistency and absolute agreement. In comparison, the data of Klink *et al.* revealed that bleb height was detected with moderate consistency and absolute agreement, whereas consistency of all other parameters were good (single ICC) or excellent (average ICC) [18].

Morphological slit-lamp biomicroscopy grading systems like the WBCS, the IBAGS or the MBCS only allow describing the surface and the superficial layers of the bleb. Even though there exist newer methods of describing filtering blebs, for example examining the internal bleb structure using ultrasound biomicroscopy [26], anterior segment optical coherence tomography [14,27-30] and in-vivo confocal microscopy [15,31,32], the WBCS remains an important tool to describe morphological appearance in clinical practice, as it is easy to use in routine practice, non-invasive and cost-efficient. Further, consistent evaluation is given by satisfactory interobserver variability as shown by Klink *et al.*[18] as well as by our data. Nevertheless, the new imaging methods might provide additional information and prognostic indicators. Also, as wound healing is modulated by the use of anti-TGFβ2 antibodies [33-35] and Mitomycin C supplemented with Cross-Linking Hyaluronic Acid [36] the use of WBCS and other classification systems remains necessary for a careful examination of the developing filtering bleb to recognize early bleb failure.

The main aim of filtering surgery is to achieve low levels of intraocular pressure in order to prevent further visual field loss [37]. Therefore, the success after TE can be measured by the achievement of a certain level of IOP. As shown in a recently-published study by Rotchford *et al.*, there exist currently 92 different IOP-based definitions of success [38]. For our study, we defined ‘success’ concerning IOP as reaching a target pressure of less or equal to 21 mmHg and IOP reduction of at least 20% of the preoperative level without any antiglaucoma medication and without any additional interventions. Using this definition of success, clinical outcomes following fornix-based TE within the last two years at our clinic were satisfying. 73.7% of our study group were classified as ‘successful’ and reached the target pressure defined above.

If additional interventions are excluded from our definition of success, the success rate increases to 77.2%. This result is comparable with other studies, which report similar success rates (between 73.9% and 76%) using the same definition of success [3,25,39].

We are aware that the ultimate goal of any bleb classification system is to predict the development of IOP over longer post-intervention periods. As agreement between WBCS and target IOP is a necessary precondition, this study only compares target IOP and WBCS at the same time to evaluate the usability and overall correlation of the WBCS and IOP in clinical practice. Therefore, no subgroup splitting according to time after surgery has been done and two examines with different levels of experience have been chosen. A larger study is ongoing to investigate the agreement of the WBCS score at early postoperative visit (<3 month and at 3 month) with the WBCS score at a later timepoint (6, 12 and 24 month). In this subsequent study we want to know if the <3 or 3 month WBCS score predicts the outcome (IOP and success) of eyes after trabeculectomy after 6, 12 and 24 month post surgery.

## Conclusion

To compare the agreement between the WBCS at <3 month and at 3 month after surgery and target IOP 6, 12 and 24 month after intervention, a follow-up study is currently in progress. In conclusion, evaluating filtering blebs after trabeculectomy by using the Wuerzburg bleb classification score is a useful tool for predicting intraocular pressure control if at least 7 points are scored. The more microcysts exist on the filtering bleb, the better the agreement with reaching the target pressure.

## Competing interests

The authors report no conflicts of interest. The authors alone are responsible for the content and writing of the paper. No financial support was received.

## Authors’ contributions

SF, MNM, JF and MTH contributed to the study design, the data analysis, interpretation, the Discussion and manuscript writing. SF, JF and MTH contributed to ophthalmologic data collection. All authors read and approved the final manuscript.

The study is registered in the registrar of the U.S. National Institute of Health (http://www.clinicaltrials.gov, NCT01287442).

## Pre-publication history

The pre-publication history for this paper can be accessed here:

http://www.biomedcentral.com/1471-2415/12/24/prepub

## References

[B1] Cairns JE Trabeculectomy, Preliminary report of a new method Am J Ophthalmol 1968 66 4 673 679 4891876

[B2] Klink T Guthoff R Grehn F Schlunck G Postoperative care after glaucoma filtration surgery Ophthalmologe 2006 103 9 815 823 quiz 824–815 16924450 10.1007/s00347-006-1404-x

[B3] Picht G Mutsch Y Grehn F Follow-up of trabeculectomy. Complications and therapeutic consequences Ophthalmologe 2001 98 7 629 634 11490740 10.1007/s003470170098

[B4] Grehn F Mauthe S Pfeiffer N Limbus-based versus Fornix-based conjunctival flap in filtering surgery, A randomized prospective study Int Ophthalmol 1989 13 1–2 139 143 2744943 10.1007/BF02028654

[B5] Lederer CM Combined cataract extraction with intraocular lens implant and mitomycin-augmented trabeculectomy Ophthalmology 1996 103 7 1025 1034 8684790 10.1016/s0161-6420(96)30571-x

[B6] Shingleton BJ Management of the failing glaucoma filter Ophthalmic Surg Lasers 1996 27 6 445 451 8782258

[B7] Vesti E Filtering blebs: follow up of trabeculectomy Ophthalmic Surg 1993 24 4 249 255 8321506

[B8] Cantor LB Mantravadi A WuDunn D Swamynathan K Cortes A Morphologic classification of filtering blebs after glaucoma filtration surgery: the Indiana Bleb Appearance Grading Scale J Glaucoma 2003 12 3 266 271 12782847 10.1097/00061198-200306000-00015

[B9] Marquardt D Lieb WE Grehn F Intensified postoperative care versus conventional follow-up: a retrospective long-term analysis of 177 trabeculectomies Graefes Arch Clin Exp Ophthalmol 2004 242 2 106 113 14648140 10.1007/s00417-003-0775-9

[B10] Picht G Grehn F Development of the filtering bleb after trabeculectomy. Classification, histopathology, wound healing process Ophthalmologe 1998 95 5 W380 W387 9643029 10.1007/s003470050285

[B11] Picht G Grehn F Classification of filtering blebs in trabeculectomy: biomicroscopy and functionality Curr Opin Ophthalmol 1998 9 2 2 8 10180508 10.1097/00055735-199804000-00002

[B12] Wells AP Crowston JG Marks J Kirwan JF Smith G Clarke JC Shah R Vieira J Bunce C Murdoch I A pilot study of a system for grading of drainage blebs after glaucoma surgery J Glaucoma 2004 13 6 454 460 15534469 10.1097/00061198-200412000-00005

[B13] Klink J Schmitz B Lieb WE Klink T Grein HJ Sold-Darseff J Heinold A Grehn F Filtering bleb function after clear cornea phacoemulsification: a prospective study Br J Ophthalmol 2005 89 5 597 601 15834092 10.1136/bjo.2004.041988 PMC1772619

[B14] Guthoff R Guthoff T Hensler D Grehn F Klink T Bleb needling in encapsulated filtering blebs: evaluation by optical coherence tomography Ophthalmologica 2010 224 4 204 208 19940526 10.1159/000260225

[B15] Guthoff R Klink T Schlunck G Grehn F In vivo confocal microscopy of failing and functioning filtering blebs: Results and clinical correlations J Glaucoma 2006 15 6 552 558 17106371 10.1097/01.ijg.0000212295.39034.10

[B16] Picht G Welge-Luessen U Grehn F Lutjen-Drecoll E Transforming growth factor beta 2 levels in the aqueous humor in different types of glaucoma and the relation to filtering bleb development Graefes Arch Clin Exp Ophthalmol 2001 239 3 199 207 11405069 10.1007/s004170000252

[B17] Wimmer I Welge-Luessen U Picht G Grehn F Influence of argon laser trabeculoplasty on transforming growth factor-beta 2 concentration and bleb scarring following trabeculectomy Graefes Arch Clin Exp Ophthalmol 2003 241 8 631 636 12898279 10.1007/s00417-003-0721-x

[B18] Klink T Schrey S Elsesser U Klink J Schlunck G Grehn F Interobserver variability of the Wurzburg bleb classification score Ophthalmologica 2008 222 6 408 413 18849624 10.1159/000161555

[B19] Sacu S Rainer G Findl O Georgopoulos M Vass C Correlation between the early morphological appearance of filtering blebs and outcome of trabeculectomy with mitomycin C J Glaucoma 2003 12 5 430 435 14520152 10.1097/00061198-200310000-00006

[B20] Funk J Frank A How can the success of a glaucoma operation be predicted? Ophthalmologe 1996 93 5 592 595 9004887 10.1007/s003470050045

[B21] Kass MA Heuer DK Higginbotham EJ Johnson CA Keltner JL Miller JP Parrish RK Wilson MR Gordon MO The Ocular Hypertension Treatment Study: a randomized trial determines that topical ocular hypotensive medication delays or prevents the onset of primary open-angle glaucoma Arch Ophthalmol 2002 120 6 701 713 discussion 829–730 12049574 10.1001/archopht.120.6.701

[B22] Leske MC Heijl A Hussein M Bengtsson B Hyman L Komaroff E Factors for glaucoma progression and the effect of treatment: the early manifest glaucoma trial Arch Ophthalmol 2003 121 1 48 56 12523884 10.1001/archopht.121.1.48

[B23] Goldmann H Schmidt T On applanation tonography Ophthalmologica 1965 150 1 65 75 5863398 10.1159/000304827

[B24] Wells AP Ashraff NN Hall RC Purdie G Comparison of two clinical Bleb grading systems Ophthalmology 2006 113 1 77 83 16389104 10.1016/j.ophtha.2005.06.037

[B25] Klink T Kann G Ellinger P Klink J Grehn F Guthoff R The prognostic value of the wuerzburg bleb classification score for the outcome of trabeculectomy Ophthalmologica 2011 225 1 55 60 20714183 10.1159/000314717

[B26] Yamamoto T Sakuma T Kitazawa Y An ultrasound biomicroscopic study of filtering blebs after mitomycin C trabeculectomy Ophthalmology 1995 102 12 1770 1776 9098276 10.1016/s0161-6420(95)30795-6

[B27] Baudouin C Labbe A El Maftouhi A Hamard P Application of anterior segment OCT to the study of glaucoma J Fr Ophtalmol 2008 31 6 Pt 2 2S5 2S9 18957906

[B28] Leung CK Yick DW Kwong YY Li FC Leung DY Mohamed S Tham CC Chung-chai C Lam DS Analysis of bleb morphology after trabeculectomy with Visante anterior segment optical coherence tomography Br J Ophthalmol 2007 91 3 340 344 17005548 10.1136/bjo.2006.100321 PMC1857643

[B29] Singh M Chew PT Friedman DS Nolan WP See JL Smith SD Zheng C Foster PJ Aung T Imaging of trabeculectomy blebs using anterior segment optical coherence tomography Ophthalmology 2007 114 1 47 53 17070581 10.1016/j.ophtha.2006.05.078

[B30] Theelen T Wesseling P Keunen JE Klevering BJ A pilot study on slit lamp-adapted optical coherence tomography imaging of trabeculectomy filtering blebs Graefes Arch Clin Exp Ophthalmol 2007 245 6 877 882 17119997 10.1007/s00417-006-0476-2

[B31] Labbe A Dupas B Hamard P Baudouin C An evaluation of blebs after filtering surgery with the in vivo confocal microscope J Fr Ophtalmol 2004 27 10 1083 1089 15687917 10.1016/s0181-5512(04)96276-6

[B32] Labbe A Dupas B Hamard P Baudouin C In vivo confocal microscopy study of blebs after filtering surgery Ophthalmology 2005 112 11 1979 16157385 10.1016/j.ophtha.2005.05.021

[B33] Cordeiro MF Role of transforming growth factor beta in conjunctival scarring Clin Sci (Lond) 2003 104 2 181 187 12546640 10.1042/CS20020150

[B34] Mead AL Wong TT Cordeiro MF Anderson IK Khaw PT Evaluation of anti-TGF-beta2 antibody as a new postoperative anti-scarring agent in glaucoma surgery Invest Ophthalmol Vis Sci 2003 44 8 3394 3401 12882787 10.1167/iovs.02-0978

[B35] Wimmer I Grehn F Control of wound healing after glaucoma surgery. Effect and inhibition of the growth factor TGF-beta Ophthalmologe 2002 99 9 678 682 12219255 10.1007/s00347-002-0698-6

[B36] Sturmer J Mermoud A Sunaric Megevand G [Trabeculectomy with mitomycin C supplemented with cross-linking hyaluronic acid: a pilot study] Klin Monbl Augenheilkd 2010 227 4 273 276 20408072 10.1055/s-0029-1245186

[B37] Group TAGIS The Advanced Glaucoma Intervention Study (AGIS): 7. The relationship between control of intraocular pressure and visual field deterioration. The AGIS Investigators Am J Ophthalmol 2000 130 4 429 440 11024415 10.1016/s0002-9394(00)00538-9

[B38] Rotchford AP King AJ Moving the goal posts definitions of success after glaucoma surgery and their effect on reported outcome Ophthalmology 2010 117 1 18 23 e13 19896196 10.1016/j.ophtha.2009.06.014

[B39] Mutsch YA Grehn F Success criteria and success rates in trabeculectomy with and without intraoperative antimetabolites using intensified postoperative care (IPC) Graefes Arch Clin Exp Ophthalmol 2000 238 11 884 891 11148811 10.1007/s004170000139

